# Exploring hospital pharmacist perspectives on ethical considerations from automated patient prioritisation: a qualitative study

**DOI:** 10.1007/s11096-026-02180-6

**Published:** 2026-07-02

**Authors:** D. Clifford, A. Wall, A. Kurdi, M. Bennie

**Affiliations:** 1 NHS Lothian, Edinburgh, Scotland; 2 Strathclyde Institute of Pharmacy and Biomedical Science, University of Strathclyde, Glasgow, Scotland; 3 College of Pharmacy, Al-Kitab University, Kirkuk, 36015 Iraq; 4 Department of Public Health Pharmacy and Management, School of Pharmacy, Sefako Makgatho Health Sciences University, Pretoria, South Africa; 5 College of Pharmacy, Hawler Medical University, Erbil, Kurdistan region, Iraq

**Keywords:** Automation, Electronic prescribing, Ethics, Health priorities, Hospitals, Pharmacists

## Abstract

**Introduction:**

The use of Hospital Electronic Prescribing and Medicines Administration (HEPMA) data in automated patient prioritisation (APP) tools is an emerging area of interest within pharmacy practice and research. As the Scottish Government continues to invest in digital transformation across health and social care, pharmacy professionals are increasingly required to adapt to new technologies whilst maintaining high standards of patient care. APP tools offer the potential to enhance operational efficiency and support clinical decision making. However, despite growing implementation internationally, there is a scarcity of published research exploring the ethical considerations associated with use.

**Aim:**

To explore hospital pharmacists’ perspectives of ethical considerations from APP tool use prior to adoption in the hospital pharmacy setting.

**Method:**

Two focus groups were conducted with a total of 12 hospital pharmacists (Pharmacist Team Manager n = 2, Advanced Pharmacist n = 5, Specialist Pharmacist n = 3, Pharmacist n = 2) from a Scottish Health Board prior to the implementation of APP using the ‘CoRE-Values’ ethical decision-making framework, with the aid of a clinical vignette for context. Focus group transcripts were analysed thematically with the four domains of the framework as mapped themes.

**Results:**

Participants considered the use of APP to be a fair and pragmatic approach to managing daily workload, enabling pharmacy teams to focus on patients most at risk of medication related harm. However, professional judgement remains essential when interpreting APP outputs, and caution must be exercised to avoid an overreliance on automation. The clinical rules underpinning APP must be regularly reviewed to ensure alignment with current practice and emerging evidence. While complete visibility of high-risk patients offers significant clinical value, it may also expose limitations in workforce capacity. Participants noted that this visibility could be leveraged to support business case development to secure further staff resource and/or service transformation.

**Conclusion:**

APP use can support clinical decision making by directing pharmacy staff to patients at risk of medication related harm in hospitals, but ethical concerns can arise if organisations do not give due consideration to these issues prior to adoption.

**Supplementary Information:**

The online version contains supplementary material available at https://doi.org/10.1007/s11096-026-02180-6.

## Impact statements

- This study highlights that APP use in hospital pharmacy raises ethical considerations that should be addressed before implementation.
- Hospital pharmacists viewed APP as a pragmatic approach to prioritising patients at greatest risk of harm from prescribed medicines but emphasised that it should complement professional judgment and be used alongside other sources of clinical information.
- The findings suggest that implementation of APP in hospital pharmacy may increase visibility of high-risk patients, exposing gaps in workforce capacity and potentially impacting staff wellbeing.

## Introduction

Healthcare systems internationally are undergoing a significant transformation, driven by digital innovation and workforce reform [1, 2]. As organisations continue to invest in digital transformation across health and social care, pharmacy professionals are increasingly required to adapt to new technologies whilst maintaining high standards of patient care. Digital innovation is central to service redesign e.g. electronic prescribing and administration systems. Various naming conventions are used internationally: ePrescribing, Electronic Prescribing and Medicines Administration (EPMA), Computerised Physician Order Entry (CPOE) and Hospital Electronic Prescribing and Medicines Administration (HEPMA) [3].

Staff recruitment and retention is a significant challenge facing healthcare organisations as workforces grow to meet increasing complexity and volume of demand; staffing shortages limit the capacity to deliver quality healthcare services and impact on staff wellbeing [2]. Due to the increased pressure on healthcare services, better use of workforce has been identified as a vital role for increased efficiency [4]. Patient prioritisation is one method used to increase efficiency and cost effectiveness of the hospital pharmacy team by directing staff resource to patients that would benefit most [4–6]. Patient prioritisation tools are already in use by pharmacists internationally to aid identification of high-risk patients, ranging from paper to electronic solutions in the hospital setting [5]. Pharmacy services with access to HEPMA system data are starting to explore automated methods of patient prioritisation.

Ethical principles have long been established in healthcare, with Beauchamp and Childress [7] first introducing a framework of four broad moral principles that have been widely adopted into medical teaching to guide decision making [8]: autonomy, beneficence, non-maleficence and justice. In the UK, pharmacists must adhere to the General Pharmaceutical Council’s (GPhC) Standards for Pharmacy Professionals [9]. Despite the growing interest in automated patient prioritisation (APP) tools, there is limited research looking specifically at the ethical considerations of use, instead focussing on clinical content and tool validation [4, 5]. The use of prioritisation tools to manage patient care must not compromise established ethical principles. Adequate staff resource may not be available to manage the provision of care to patients deemed a priority and it is therefore important to explore pharmacist views on using APP in the hospital pharmacy context prior to implementation.

## Aim

To explore hospital pharmacists’ perspectives of ethical considerations from APP tool use prior to adoption in the hospital pharmacy setting.

## Method

### Study design and setting

A qualitative study design was used to explore the study aim and reported using the Standards for Reporting Qualitative Research (SRQR) [10]. Two in person focus groups utilising a semi-structured topic guide were conducted to explore pharmacist opinion [11–13].

This study was set within National Health Service (NHS) Lothian, which has an implemented HEPMA system but did not use APP during this study. NHS Lothian is a Scottish Health Board that provides primary, community, acute and tertiary hospital services to over 850,000 people [14]. Since July 2020, the HEPMA system has been implemented across 3398 (93%) inpatient beds across the Health Board.

### Participants

Participants were registered hospital pharmacists involved in inpatient pharmaceutical care and/or management of inpatient clinical pharmacy teams. Recruitment was by purposive convenience sampling [15] through the NHS Lothian Clinical Pharmacy Group (comprised of clinical service leads and site representative clinical pharmacists) and Pharmacy Leadership Team. At least 12 pharmacists were recruited to achieve a minimum of 6 participants per focus group, an adequate number for qualitative content generation in thematic analysis [16, 17].

Pharmacists of all NHS bands were invited by e-mail and senior staff (Advanced Pharmacists and Pharmacist Team Managers) were separated from junior staff (Specialist Pharmacists and Pharmacists) in the focus groups to promote candid participation. Volunteers were invited to a 30-min virtual briefing session (on Microsoft Teams®), which demonstrated potential APP tool functionality and allowed participants to clarify understanding. Participants were given a clinical vignette (Appendix 1) at the end of the briefing session detailing an example of what an APP tool could display to a pharmacist on a given day. Consent via e-mail was mandatory prior to focus group attendance.

### Research materials

The clinical vignette was developed with a pharmacist project supervisor to frame discussion around the use of an APP tool [18, 19].

**Fig. 1:**
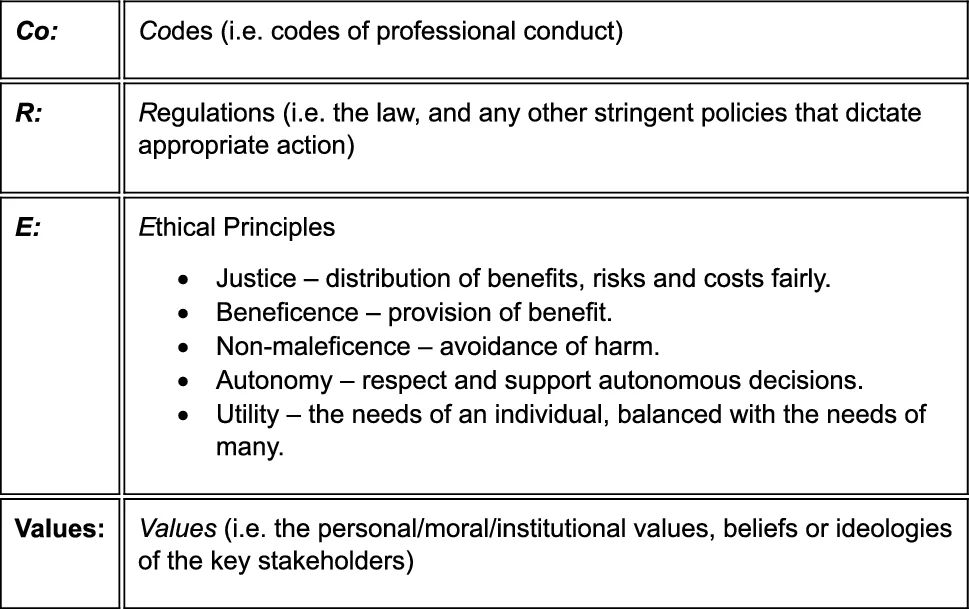
CoRE-Values framework [8]

A focus group topic guide (Appendix 2) was created using the CoRE-Values ethical framework [8], comprised of four domains; Codes (Professional); Regulation; Ethical principles and Values (Fig. 1).

Questions were developed using the domain definitions in collaboration with a pharmacist project supervisor. Each question was framed around the domain or principle definition to prompt discussion on each topic in relation to APP use. The framework was selected as it incorporates the four established principles of medical ethics in addition to the context necessary for ethical decision making and can aid the systematic identification and consideration of ethical aspects in clinical cases [8]. The documentation was piloted with a lead clinical pharmacist (recruited to the study) who met the inclusion criteria and had background knowledge of the APP tool function.

### Data collection and analysis

In person focus groups were held one week after the briefing session (October 2024) and conducted on the same morning, led by the lead researcher (an experienced hospital pharmacist and HEPMA system specialist) with an assistant moderator (HEPMA pharmacist). Written consent was confirmed and pseudo anonymity clarified by the lead researcher i.e. participant names were not included within the focus group transcripts or manuscript. Focus group discussions were audio recorded using a Dictaphone® with 256-bit file encryption and the clinical vignette was displayed to the participants for reference as the topic guide questions were asked. The GPhC Standards for Pharmacy Professionals were also displayed to participants when discussing codes of professional conduct [9]. Audio recordings were stored in a secured shared drive to maintain confidentiality and the recordings were deleted from the Dictaphone®. Participants were given a unique identifier on a participant key by the lead researcher and the audio recordings transcribed to Microsoft Office Word® documents using an intelligent verbatim approach, stored in a secured shared drive. The first focus group transcript was independently reviewed against the audio recording by a HEPMA pharmacist to check transcription accuracy.

Participant demographic information (gender, age, job title and NHS banding, number of wards covered, managerial responsibility, employment contract and years of experience) was collated in a Microsoft Excel® spreadsheet. Transcripts were analysed thematically by the lead researcher using the steps outlined by Braun and Clarke [20–22]. A deductive approach to initial analysis was taken due to the use of a pre-existing framework and the transcript text was mapped to the four framework domains as themes. Subthemes were identified inductively for two domains (Regulations and Values) as the framework did not contain domain subthemes or have associated standards that could be applied, and deductively for two domains that contained subthemes (Codes (Professional) and Ethical Principles). The Codes (Professional) domain was analysed using the nine standards of the GPhC’s Standards for Pharmacy Professionals [9] (outlined in Table 1) as the regulatory body for pharmacy staff in the UK and the ethical principles domain was analysed using the five ethical principles within the domain: Justice, Beneficence, Non-maleficence, Autonomy and Utility. The first focus group transcript was independently analysed by a University of Strathclyde research student and coding compared for consistency.

**Table 1:** General pharmaceutical council (GPhC) professional standards [9] used as subthemes for the Codes (Professional) theme analysis

Standard	Description
1: Person-centred care	Person-centred care is delivered when pharmacy professionals understand what is important to the individual and then adapt the care to meet their needs–making the care of the person their first priority
2: Partnership working	A person’s health, safety and wellbeing are dependent on pharmacy professionals working in partnership with others, where everyone is contributing towards providing the person with the care they need
3: Effective communication	Effective communication is essential to the delivery of person-centred care and to working in partnership with others. It helps people to be involved in decisions about their health, safety and wellbeing
4: Professional knowledge and skills	A pharmacy professional’s knowledge and skills must develop over the course of their career to reflect the changing nature of healthcare, the population they provide care to and the roles they carry out
5: Professional judgement	People expect pharmacy professionals to use their professional judgement so that they deliver safe and effective care. Professional judgement may include balancing the needs of individuals with the needs of society as a whole
6: Professional behaviour	People expect pharmacy professionals to behave professionally. This is essential to maintaining trust and confidence in pharmacy
7: Privacy and confidentiality	Maintaining confidentiality is a vital part of the relationship between a pharmacy professional and the person seeking care
8: Speaking up about concerns	The quality of care that people receive is improved when pharmacy professionals learn from feedback and incidents, and challenge poor practice and behaviours. This includes speaking up when they have concerns
9: Effective leadership	Wherever a pharmacy professional practises, they must provide leadership to the people they work with and to others

### Ethics approval

The Strathclyde Institute of Pharmacy and Biomedical Science (SIPBS) Ethics Committee approved the study on the 23rd of May 2024 and the NHS Lothian Research and Development office approved the study in July 2024 (IRAS Project ID 345885).

## Results

Twelve pharmacists participated across a range of NHS pharmacist bands (Pharmacist to Pharmacist Team Manager), with an even split of genders. Most participants were Advanced Pharmacists that worked full time in more than one ward. Participant characteristics are displayed in Table 2.

**Table 2:** Participant demographics (n = 12)

Demographic		n
Gender	Female	6
	Male	6
Age [years]	20–29	5
	30–39	2
	40–49	3
	50–59	2
Pharmacist title and NHS banding	Pharmacist–P (AFC* Band 6)	2
	Specialist Pharmacist–SP (AFC* Band 7)	3
	Advanced Pharmacist–AP (AFC* Band 8a)	5
	Pharmacist Team Manager–PTM (AFC* Band 8b)	2
Number of wards covered by pharmacist	0	1
	1	3
	2	5
	3	1
	4	2
Managerial responsibility for clinical service	Yes	6
	No	6
Employment	Full time	11
	Part time	1
		Median (IQR)
Years’ experience	Working in current role	2.9 (1–3)

^*^AFC – NHS Agenda for change banding

**Fig. 2:**
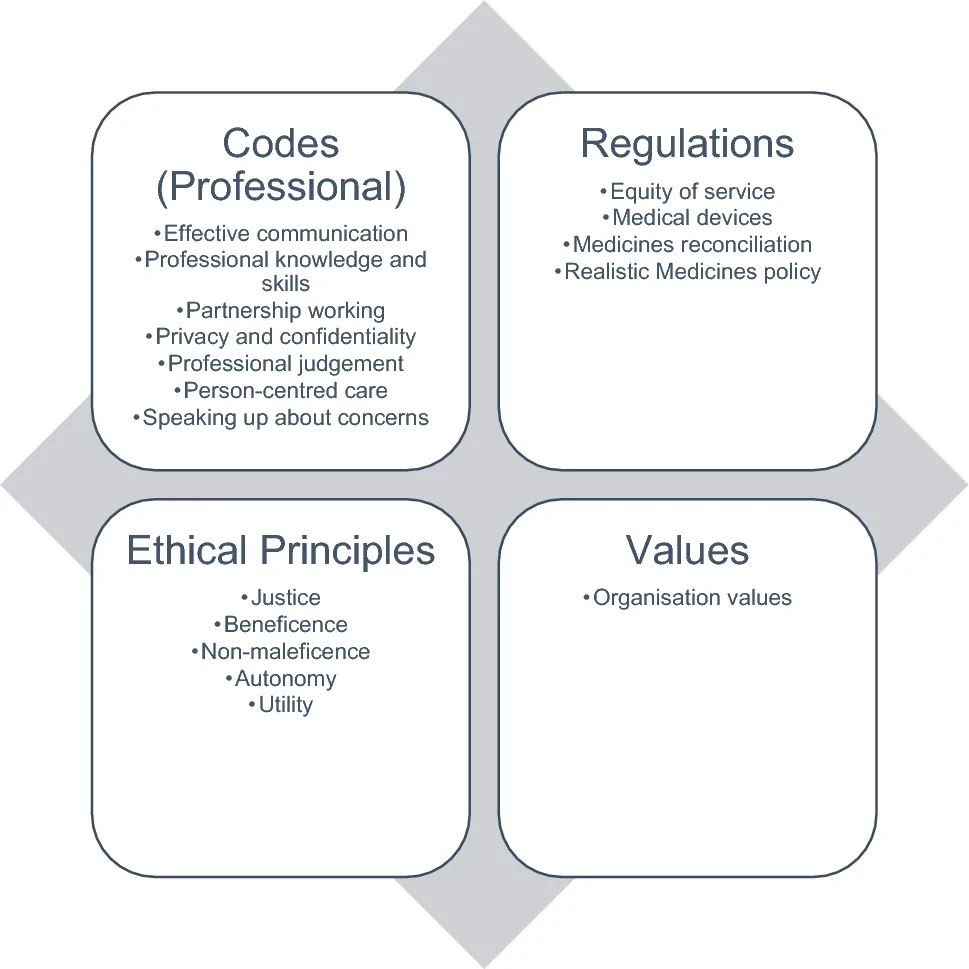
Summary of subthemes discussed for each CoRE-Values framework domain

The four CoRE-Values ethical framework domains [8] were used to structure the data analysis of the focus groups to systematically examine ethical considerations of APP tool use. A summary of the theme content and identified subthemes are summarised in Fig. 2.

### Theme 1: codes (professional)

Seven subthemes (Fig. 2) were identified from the nine GPhC professional standards (Table 1).

Effective communication through ward presence and nurse interaction remains essential to capture patient information not contained within an APP tool:

“*I think that's why it's important, although it's a tool, to still have effective communication with the nurses by doing morning huddles*” (P4 - AP)

Participants also highlighted that overreliance on automation could undermine professional knowledge and skills in patient prioritisation: 

*“…pharmacists from qualification level upwards who are reliant on a patient prioritisation tool, they perhaps are not going to use their clinical judgement... So, I think that… could be presented as a risk…*” (P7 - AP)

Partnership working was thought to be a challenge and a benefit of APP use, as multidisciplinary team priorities may conflict with APP, but it could also assist with pharmacy team working across wards and services.

“*…when you are on the ward you might get pulled in different directions from the nursing staff and the medics… and that might be a patient that from your tool [APP] would be medium risk and it wouldn’t be on your high risk…*” (P11 - SP)

“*…better partnership working across the clinical pharmacy team…*” (P9 - SP)

The need for adequate data protection when automatically using patient data for prioritisation was emphasised to ensure patient privacy and confidentiality:

“*…make sure that we are using automated systems like this… in line with GDPR…*” (P9 -SP)

Participants agreed that APP should complement, not replace, professional judgement. Risk that pharmacy staff could use automated risk scores without applying clinical judgement to assess all patient risk factors was acknowledged, but it was suggested that this could be mitigated through APP training: 

“*…I guess if there's automation coming in… it's maybe taking the edge off that, doesn’t allow you to use your judgement.*” (P2 - AP)

APP was also viewed to support patient centred care by helping to identify high‑risk patients and target pharmacy resources to them:

“*…is facilitating the pharmacy team towards the high-risk patients, which with the current system we potentially miss or we do miss*” (P7 - AP)

Participants also felt that visibility of high-risk patients could drive requests for additional pharmacy resources as staff speak up about capacity concerns.

“*…we've not got capacity, is there any way to try… and reduce the risk of harm through investment in services*” (P7 - AP)

### Theme 2: regulations

Four subthemes emerged (Fig. 2) when participants considered regulatory aspects of using APP. APP models, which prioritise high-risk patients for pharmaceutical intervention, were considered to not fully align with any NHS policy that emphasises equitable access to person centred care. There was a perception that low-risk patients may not routinely be reviewed by pharmacy staff. However, not all participants felt this was the case as clinical pharmacists do not routinely see every patient currently:

“*I don’t think it’s really in… conflict… the point [P7] made, obviously that’s in… an ideal situation where we can see every single patient… we know that's not going to happen ever because we're not a 24/7 service.*” (P6 - PTM)

A question was posed if APP tools using HEPMA system data are classed as a medical device and if so, what are the implications from a legislative perspective in using them within an organisation:

“*thinking about other electronic tools and registered medical devices… if you're using a tool like this [APP]… does that open you to a risk from an organisational perspective*” (P7 - AP)

One participant suggested there could be conflict with the Scottish Government’s goal of conducting 95% of medicines reconciliations (med rec) within 24 h of patient admission. However, most participants did not feel that this would be an issue as the number of patients reviewed by pharmacists would not change when using APP.

“*I don’t think using the tool [APP] would stop you meeting the NHS standards for… patients med rec’d in 24hrs… at the moment, we don’t… meet the standard.*” (P9 - SP)

APP was also viewed to assist pharmacy staff by delivering care in line with the NHS Scotland Realistic Medicine policy.

“*I think we would want to be reviewing every patient, but I don’t think we need to review every patient and this tool [APP] would allow us to see the right patient at the right time…deliver in line with the realistic medicine policy.*” (P9 - SP)

### Theme 3: ethical principles

The five subthemes (Justice, Beneficence, Non-maleficence, Autonomy and Utility) from the ethical principles domain of the CoRE-Values framework identified several participant concerns.

Participants highlighted that “*…we should have the same service for patients regardless of background…*” (P7 - AP) but “*…if you have a finite amount of resource you have to focus it on the right patients*” (P1 - PTM). There was some concern with the automated approach as “*…directing your clinical service towards this model… you've got a low risk cohort that's defined… that might not get the same quality*” (P7 - AP) of care. One participant felt there was a potential solution to this problem through staff skill mixing and allocating “*…patients based on the professional role… with pharmacy support workers and the technicians*” (P1 - PTM). Overall, participants thought that “*…it’s actually more equitable to use something like this* [APP] *to… direct*” (P5 - AP) pharmacy staff to patients most at risk of harm from prescribed medications. APP would “*…increase the equity of care because you’re… able to see the correct patients*” (P9 - SP) as “*…right now we are reviewing some patients to rule out that they don’t need seen*” (P8 - P).

Automatically prioritising patients was felt to “*…take a bit of the subjectivity out… as a starter*” (P5 - AP) when assessing ward patients for review as risk level is not known until patient medications are reviewed. Automation of prioritisation “*…would target the pharmacist to the right patient*” (P1- PTM), particularly if staff resource is suboptimal. Highlighting patients on high-risk medicines in high turnover wards was felt to be beneficial as “*…that's where… you're likely to miss the high-risk patients…*” (P6 - PTM) due to patient transfer through hospital. Limited benefit was expressed for specialties with numerous patients on high-risk medicines “*like oncology potentially, paediatrics potentially, transplant.*” (P7 - AP).

Automation will support harm reduction by highlighting patients on high-risk medicines:

“*I think harm will be reduced… because the high-risk meds is probably where the Datix* [incident reports] *are most likely to be seen*” (P4 - AP)

However, high-risk patient visibility and knowledge of this through automated prioritisation was felt to be “…*an informed worry*” (P12–P) and was highlighted as a concern if an individual or team is unable to review each high-risk patient within working hours. There was concern that patients would be visible in wards without clinical pharmacist input and what the outcome of this would be, it is “*the ethical side of being aware that you have got… high-risk patients in a ward that’s not got a clinical pharmacy service*” (P9–SP). Participants also discussed concerns around the data used to prioritise patients automatically as outlined in Table 3. It was proposed that APP tools should be used in conjunction with other sources of patient information by clinical pharmacy staff when prioritising patients to ensure informed decisions are made.

**Table 3:** Participant concerns with data used to automatically prioritise patients

Concern	Description	Illustrative quote
Omitted medications	Medication not prescribed on HEPMA system through omission will not be included within automated prioritisation	*“…if a medicine has been missed off HEPMA, it* [automated patient prioritisation] *won’t catch that and therefore a patient could be high risk” (P4–AP)*
Not all patient clinical factors are included	Patient blood results, weight or health conditions may not be included with the automated prioritisation	*“…clinical status might change that’s not picked up pharmaceutically…deteriorating renal function for instance” (P2–AP)*
Maintenance of prioritisation rules	Need for continued update of the rules that govern the automated prioritisation	*“We need to continually just check it’s* [automated patient prioritisation]* not missing something” (P1–PTM)*

Participants acknowledged that there is potential for autonomy to be taken away from clinical pharmacists:

“*…it does take away from your autonomy I feel… because it really directs you… you’re gonna look at… red* [high risk] *patients instead of… green ones* [low risk].” (P10 - P)

However, APP was thought to supplement the information that clinical pharmacists use to prioritise patients:

“*it supports, not replaces your clinical judgement of the hospital pharmacist or technician*” (P7 - AP)

It was thought necessary to state “… *in the induction and the rollout very clearly*” (P1–PTM) the continued need to use additional sources of patient information when making decisions on patient prioritisation and to have transparency of the automated prioritisation score for patient prioritising when capacity is limited within the working day. Furthermore, participants discussed how APP could be used to transform a clinical pharmacy service model to utilise resources effectively:

“*Currently our model is… specialty based… ward based…do we start to think… actually it's more fluid. Where pharmacists are going to the areas where this tool* [APP] *is helping to identify high-risk patients*” (P6 - PTM)

### Theme 4: values

The perceived values and beliefs of participants were explored to identify areas of synergy or potential conflict. Organisational values was the only subtheme identified. One participant thought that use of automation in patient prioritisation would align with “*the pharmacy strategy and… strategic plan…our values as an NHS Board and organisation*” (P7–AP) as it is an innovation to support patient care.

## Discussion

This study aimed to explore hospital pharmacists’ perspectives of ethical considerations from APP tool use prior to adoption in the hospital pharmacy setting.

### Statement of key findings

Ethical issues with APP can arise if pharmacy staff resource cannot review all high risk patients identified by APP; if APP is used to prioritise patients without professional judgement there is risk that not all patient clinical information will be reviewed, and staff may lose autonomy; if APP tools are not updated to reflect current practice then there is risk of patient harm. Despite ethical concerns, use of APP brings opportunity for service evaluation and transformation.

### Interpretation

APP may introduce ethical conflict if not managed during implementation. The four key themes of potential ethical concerns are presented under two headings due to overlap and interrelation.

### Codes (professional) and ethical principles

The requirement to use APP alongside other patient information sources to assess patient risk when prioritising for pharmaceutical review was identified as necessary by participants in this study as APP using solely HEPMA system data may not contain all of the clinical information required by pharmacists to prioritise patients. This is dependent on the clinical rules created for APP and software available locally with interoperability to link data sets, reinforcing the importance of the tool design and development. Variability in the criteria underpinning APP tools in hospital pharmacy is demonstrated in the literature, with published tools differing in target patient populations, patient risk factors and use of laboratory data [4–6]. This variation underscores the importance of pharmacists maintaining an understanding of the criteria embedded within APP tools they use, while also ensuring they supplement these tools with broader clinical information. In practice, this includes actively considering patient specific risk factors that may not be captured within locally agreed APP rules, thereby supporting more comprehensive and safe clinical decision making.

Participants were concerned that APP may identify more high-risk patients than the existing pharmacy resource could review within working hours. This unintended consequence of automated prioritisation has to our knowledge not been identified in the literature and is a potential ethical concern highlighted by participants in this study. Published literature on APP tools instead focuses on tool content, validation and implementation [4–6]. Hospital pharmacy services should consider this prior to implementation as it may highlight high-risk patients in areas with little or no pharmacy service leading to requests for increased resource or modified service delivery models. Staff wellbeing could also be impacted if all identified high-risk patients across the pharmacist’s assigned ward(s) cannot be reviewed during the working shift or day, individuals may experience moral distress.

However, use of APP could change traditional pharmacy service models by directing pharmacy resource to high-risk patients across a specialty or hospital rather than within defined ward(s) as described by Cottrell et al. [23]. Taking a skill mix approach to managing the different patient priority levels of APP (low, medium and high risk) through skill utilisation of the clinical pharmacy technician for lower priority patient review as suggested in this study was reported by Abuzour et al. [4], necessitating clear roles and responsibilities when utilising APP tools. Targeting pharmacy resource to high-risk patients through patient prioritisation tool use is acknowledged as a pragmatic approach intended to improve service efficiency and support patient safety [4, 5, 23].

The risk in solely using HEPMA data for APP is a recognised limitation [23, 24]; any omitted patient medication will not be included within APP scoring and therefore the assigned risk score could be misrepresentative if this occurs. Similarly, there will be a need for ongoing clinical rule update to ensure new high-risk medicines are included. The need for continued tool management to keep up to date with recommended practice was recognised by Levivien et al. [25].

### Regulations and values

The concern that APP use could be in conflict with NHS policy could be mitigated through efficient use of resources by targeting patients most in need of care [26], and the uncertainty raised around APP qualifying as a medical device with regulatory implications for organisations adopting such tools has not been clearly addressed in the literature. This may reflect variation in APP use and individual interpretation of the regulatory guidance, which has continued to evolve over time with the increasing interest in artificial intelligence for healthcare. The Medicines & Healthcare products Regulatory Agency in the UK have published guidance to assist organisations in assessing such tools [27].

The Royal College of Pharmacy in the UK have set out their vision for 2030 and use of data, digital technology and innovation are listed as key elements of an infrastructure to underpin the vision [28]. Use of clinical data to target pharmacy interventions and prioritise patients with risk factors is identified as a necessary enabler. APP use in hospital pharmacy is therefore broadly aligned with healthcare organisational values and a future vision for pharmacy in the UK. Leadership bodies should however recognise the potential challenges to APP implementation based on the ethical concerns presented in this article. The four organisational values upheld by NHS Scotland–care and compassion; dignity and respect; openness, honesty and responsibility; and quality and teamwork [29] – appear compatible with APP. However, values may differ between healthcare organisations and should be reviewed prior to APP adoption to ensure no conflicts are identified.

### Strengths and limitations

Ethical consideration of APP use in hospital pharmacy has not been published prior to this study to the authors’ knowledge. Incorporating an ethical decision-making framework was a strength of the study design, providing a structured approach to data analysis and supporting the transferability of the results. However, its’ evaluation was previously confined to a teaching context with nursing students, medical students and instructors, which may limit broader applicability. As the study was conducted within a single Scottish Health Board, this may limit the transferability of the findings, as other healthcare organisations may have differing staffing models, HEPMA systems or patient prioritisation processes.

Pharmacy technicians were not recruited to this study but were referenced in the focus groups, this was therefore a limitation as not all staff groups who may use APP in pharmacy were included. Conducting only two focus groups did not achieve full data saturation, but this approach can identify 80% of themes [30]. Due to recruitment challenges, focus group number is a limitation of this study. Furthermore, the research materials were piloted with a pharmacist team manager only and not with pharmacists from a range of grades, which could have been a limitation.

### Further research

Future work on ethical considerations should be conducted with an organisation that has APP established in hospital pharmacy practice. As APP use evolves with machine learning models and artificial intelligence, further research is necessary around tool validation and assessing patient outcomes; a limitation noted within the literature [31]. Further work on transformational change from APP use and staff wellbeing is warranted to fully understand the post implementation impact of APP utilising HEPMA system data.

## Conclusion

Overall, pharmacist participants viewed APP as a positive innovation that would enhance patient safety through effective management of staff resources by directing hospital pharmacy staff to patients at greatest risk of medication related harm. APP was also perceived to bring opportunity by facilitating transformational change of a hospital pharmacy service model through a more centralised approach. However, this study highlights that ethical concerns may arise if organisations do not give due consideration to these issues prior to adopting APP tools in hospital pharmacy practice.

## Supplementary Information

Below is the link to the electronic supplementary material.

Supplementary file1 (DOCX 111 KB)

Supplementary file2 (DOCX 44 KB)

## Data Availability

The data that support the findings of this study are available from the corresponding author upon reasonable request.
