# Supplementary material for: Exploring hospital pharmacist perspectives on ethical considerations from automated patient prioritisation: a qualitative study
Source: Int J Clin Pharm. 2026 Jul 2;48(5):2005–14. doi: 10.1007/s11096-026-02180-6 (PMC13569551; doi:10.1007/s11096-026-02180-6)
Supplement: Supplementary file 1 [file 11096_2026_2180_MOESM1_ESM.docx]

**Clinical Vignette**

This clinical scenario is to be read in advance of focus group attendance. During the focus group, you will be asked questions based on the CoRE-Values ethical framework to prompt discussion around the use of automated patient prioritisation tool use with a Hospital Electronic Prescribing and Medicines Administration (HEPMA) system as described in the scenario below.

The CoRE-Values framework comprises of four domains:

| ***Co*:** | = | *Co*des (i.e. codes of professional conduct) |
| --- | --- | --- |
| ***R*:** | = | *R*egulations (i.e. the law, and any other stringent policies that dictate appropriate action) |
| ***E*:** | = | *E*thical Principles |
| **Values:** | = | *Values* (i.e. the personal/moral/institutional values, beliefs or ideologies of the key stakeholders) |

Please familiarise yourself with the GPhC standards for pharmacy professionals prior to the focus group, which can be found here: [standards_for_pharmacy_professionals_may_2017_0.pdf (pharmacyregulation.org)](https://www.pharmacyregulation.org/sites/default/files/standards_for_pharmacy_professionals_may_2017_0.pdf).

The automated patient prioritisation tool included within this scenario is described in the briefing session.

**Scenario**

- It is a Monday morning, the pharmacy patient prioritisation tool is automatically updated for a hospital of 600 in-patients. In this hospital, 75% of in-patient wards receive a routine clinical pharmacy service.
- The patient prioritisation tool displays 200 high risk patients across the hospital, some in wards without a clinical pharmacy service. The tool also highlights 250 medium risk patients and 150 low risk patients.
- As a ward pharmacist, you have 1 session (3.75hrs) to cover two wards of 30 in-patients that have 16 high risk patients identified, 24 medium risk and 20 low risk.
