# Supplementary material for: Exploring hospital pharmacist perspectives on ethical considerations from automated patient prioritisation: a qualitative study
Source: Int J Clin Pharm. 2026 Jul 2;48(5):2005–14. doi: 10.1007/s11096-026-02180-6 (PMC13569551; doi:10.1007/s11096-026-02180-6)
Supplement: Supplementary file 2 [file 11096_2026_2180_MOESM2_ESM.docx]

**Focus Group Topic Guide**

**Lead investigator to confirm all participants attended mandatory briefing session as well as completing the consent and demographic forms prior to focus group.**

The aim of this focus group is to explore your views on ethical considerations in using an automated patient prioritisation tool with a HEPMA system.

Thank you for agreeing to participate in this focus group today. Please be aware that after this meeting, participant data will not be able to be withdrawn but all data will be anonymised.

Is everyone still happy to proceed?

Today’s focus group will be audio recorded. Does anyone have any questions before we begin the recording?

| Activity | Time (mins) |
| --- | --- |
| **Introduction** | **5** |
| - Introduce self. - Brief rationale for focus group: part of MPhil project. - Ask participants to introduce themselves by providing their first names and clinical practice area. |  |
| **Focus Group Agreements** | **2.5** |
| - No right or wrong answers. - Feel free to confidently speak your thoughts, listen to others with attention and please be respectful of each other. - This is a safe space to provide your opinions. Anything you say within the focus group will be treated confidentially. |  |
| **Questions for Discussion** | **50** |
| CoRE Values Framework  Domain 1  **Co**des of Professional Conduct   - What are the potential concerns (if any) in using automated patient prioritisation as described in the vignette with respect to the General Pharmaceutical Council (GPhC) standards for pharmacy professionals?   Domain 2  **R**egulations   - What (if any) conflicts are there with law or NHS policy in using the automated patient prioritisation tool?   Domain 3  **E**thical Principles   - Justice – What is the impact of using an automated patient prioritisation tool on the fairness and equity of the delivery of pharmaceutical care. - Beneficence – How do you think provision of benefit is applied by using automated prioritisation in the vignette. - Non-maleficence – How would harm be avoided by using automated patient prioritisation in the vignette? - Autonomy – What impact does automated patient prioritisation have on the ability of hospital pharmacists to make reasoned choices? - Utility – How do you think automated patient prioritisation balances the needs of high risk to low risk patients in the vignette?   Domain 4  **Values**   - What are your personal concerns (if any) as a health professional in using an automated patient prioritisation tool as described in the vignette? |  |
| **Closing** |  |
| - Thank everyone for participating in focus group. - Explain that participants can contact the lead investigator for any updates. | **2.5** |
